# Frequent Surfing on Social Health Networks is Associated With Increased Knowledge and Patient Health Activation

**DOI:** 10.2196/jmir.5832

**Published:** 2016-08-10

**Authors:** Dafna Grosberg, Haya Grinvald, Haim Reuveni, Racheli Magnezi

**Affiliations:** ^1^ Bar Ilan University Interdisciplinary Department for Social Sciences Ramat Gan Israel; ^2^ Bar Ilan University Public Health and Health Systems Management Program Ramat Gan Israel; ^3^ Ben Gurion University of the Negev Department of Health Policy and Management Beer Sheva Israel

**Keywords:** Internet, social networks, social media, pain, Patient Activation Measure (PAM), chronic disease, diabetes mellitus

## Abstract

**Background:**

The advent of the Internet has driven a technological revolution that has changed our lives. As part of this phenomenon, social networks have attained a prominent role in health care. A variety of medical services is provided over the Internet, including home monitoring, interactive communications between the patient and service providers, and social support, among others. This study emphasizes some of the practical implications of Web-based health social networks for patients and for health care systems.

**Objective:**

The objective of this study was to assess how participation in a social network among individuals with a chronic condition contributed to patient activation, based on the Patient Activation Measure (PAM).

**Methods:**

A prospective, cross-sectional survey with a retrospective component was conducted. Data were collected from Camoni, a Hebrew-language Web-based social health network, participants in the diabetes mellitus, pain, hypertension, and depression/anxiety forums, during November 2012 to 2013. Experienced users (enrolled at least 6 months) and newly enrolled received similar versions of the same questionnaire including sociodemographics and PAM.

**Results:**

Among 686 participants, 154 of 337 experienced and 123 of 349 newly enrolled completed the questionnaire. Positive correlations (*P*<.05) were found between frequency and duration of site visits and patient activation, social relationships, and chronic disease knowledge. Men surfed longer than women (χ²_3_=10.104, *P*<.05). Experienced users with diabetes surfed more than those with other illnesses and had significantly higher PAM scores (mean, M=69.3, standard deviation, SD=19.1, PAM level 4; Z=−4.197, *P*<.001) than new users (M=62.8, SD=18.7, PAM level 3). Disease knowledge directly predicted PAM for all users (β=.26 and .21, respectively). Frequency and duration of social health network use were correlated with increased knowledge about a chronic disease. Experienced surfers had higher PAM than newly enrolled, suggesting that continued site use may contribute to increased activation.

**Conclusions:**

Web-based social health networks offer an opportunity to expand patient knowledge and increase involvement in personal health, thereby increasing patient activation. Further studies are needed to examine these changes on other aspects of chronic illnesses such as quality of life and costs.

## Introduction

According to Pew [1] and Harris [2] polls, more than 70% of Internet users looked on the Internet for health information. Across all age groups, Internet use has increased from 2005 through 2013 [3]. Individuals 18 to 29 years of age are most likely to use social networking sites (89%), and men and women surf equally among them [1].

Patients with chronic illness are less likely to use the Internet, although use is still high at 62% [4]. Moreover, research indicates that Internet users with a chronic illness are more likely to blog or contribute to a Web-based discussion, a listserv, or other Web-based group forum that helps people with personal issues or health problems [5,6].

Patient activation describes how much a patient is involved in his or her health care. It includes self-efficacy, behavior, and knowledge and can predict healthy behaviors, preventive care measures, disease-specific self-care behavior, and information seeking [7]. Activated patients are better at self-management [8-10], which affects health outcomes [11], decreases health care costs, and increases quality of life [12].

The Internet can impact self-management and activate patients. Patient activation varies by socioeconomic status [6,13], and the Internet offers a potential mechanism to reduce these inequalities. Studies include Web-based self-management [14], patient portals [15,16], Web-based patient activation interventions [17], and Web-based interventions for a variety of health-related issues ranging from chronic conditions, health promotion, and mental health. Overall, there have been small, but statistically significant effects [18]. A review of studies of chronically ill samples and the impact of participation in Internet programs that combine health information with Web-based peer support, decision support, or help with behavior, found improvements in users’ knowledge, social support, health behaviors, and clinical outcomes. Participants’ self-efficacy was enhanced as well [19,20]. In addition, eHealth, Web-based interventions provide cost-effective patient empowerment or at least suggest promising evidence thereof [21].

Little is known about Web-based social networks. A review on the impact of Web-based social networks on health behavior changes concluded that there is very modest evidence that interventions incorporating Web-based social networks may be effective [22,23].

We previously found that a social health network (without interventions) led to increased patient activation, as active participants serve as role models for other participants for health management [24]. They learn self-efficacy, gain knowledge, and learn to behave in ways consistent with self-management. In this study, we examined the association of continued participation in a social health network with patient activation after 3 months of enrollment.

## Methods

### The Platform

The research was conducted using the Camoni (which means “Like me” in Hebrew) site, which was established in 2008. Camoni is the first Hebrew-language social network. It is targeted to individuals with chronic conditions and it helps them find others facing similar health issues.

The Camoni site is comprised of 16 communities (diabetes mellitus, chronic pain, heart disease, hypertension, obesity, eating disorders, multiple sclerosis, spinal injury, lung disease, kidney disease, stroke, osteoporosis, Crohn’s disease, cancer, obesity, and depression), defined according to chronic health conditions. A medical expert (physician, nurse, or psychologist) heads each community. Camoni offers advice, the opportunity to consult with experts, and the chance to converse with other patients who face the same health condition. The site includes blogs, forums, support groups, internal mail, and chats. It also explains each health condition, its diagnosis, and offers practical advice on how to maintain one’s health and cope with the disease. Registration is required for active participation on the site, which is open to all. More than 21,000 patients have registered, and 400 to 500 join monthly.

### Recruitment

This was a cross-sectional survey with a retrospective element. Data were collected from November 1, 2012, to October 31, 2013. The study focused on the 4 largest Camoni communities: diabetes mellitus (n=5015), pain (n=4255), depression (n=3877), and hypertension (n=3821). Eating disorders were not included despite its size (n=4500) due to community sensitivities. The respondents were divided into 3 groups. The first group, defined as “newly enrolled,” was comprised of those who had just joined the site. The second group defined as “experienced respondents,” was comprised of those who had used the site for at least 6 months before the beginning of the study. The third group included a subgroup defined as “experienced and new respondents” who answered the initial questionnaire and a follow-up approximately 3 months after the registration. The study was approved by the Ethics Committee of Bar Ilan University.

### Instruments

Two similar versions of the same questionnaire were used. The first was for experienced users and included 49 questions, written in the present tense. For example, “How much support are you getting from the site” The second questionnaire was for the newly enrolled respondents and included 48 questions, written in the future tense. For example, “How much support do you expect to get from the site?” The questionnaire was sent to everyone who registered at the site during the study period. Both questionnaires queried sociodemographics, the self-reported effect of the use of the site, and personal involvement in the chronic illness.

The questionnaire was accessible through Google Docs. An invitation to participate and a link were available on the Camoni home page of each of the 4 disease communities. During the recruitment period, reminders were placed in the monthly newsletter sent to all Camoni respondents who had not declined the option.

### Demographic and Health Characteristics

Respondents were asked to provide information about basic demographic variables such as age, gender, education, family status, income, and diagnosis, as well as information regarding their use of the website.

### Personal Involvement in Health Care Related to Site Use

This section was based on the work of Lemire et al [25]. It measures the site's influence on respondents' health behaviors. Two similar versions were used. The questionnaire for veteran users included 20 items, and the questionnaire for the new respondents included 17 items. The number of questions for each group differed because some questions were not relevant to new users. For example: “How frequently do you visit the site on average?” Possible responses ranged from “several times a day” to “less than once a month”; “About how long do you spend on the site each time?” Possible responses ranged from “less than 10 minutes” to “more than an hour”; and “Are you an active user of the site (write or post notices, tips, etc.)?” with a “yes” or “no” response. Both versions used a 4-point scale (1=strongly disagree to 4=strongly agree). There were no reversed items. The items were divided into 3 dimensions: (1) Knowledge about the disease—for example, “The site allows me to develop a better understanding of my personal health” (4 items, α=.846); (2) Social relationships—for example, “The site helps me feel less lonely” (4 items, α=.811); and (3) Involvement in personal health—for example, “The site makes me feel more conﬁdent about the choices I plan on making” (10 items, α=.916).

### Patient Activation Measure (PAM)

This section was based on the work of Hibbard et al [26]. It captures the degree to which patients have the beliefs, knowledge, and skills to manage their condition(s), collaborate with their providers, maintain their health, and access appropriate and high-quality care [27]. The PAM includes 13 items on a 4-point scale (1=strongly disagree to 4=strongly agree). An interval scale of 0 to 100 was calculated (Cronbach α=.899). The interval score can be divided into 4 levels. Level 1: May not yet believe that the patient role is important (PAM score of 45.2 or lower); Level 2: Lacks confidence and knowledge to take action (PAM score of 47.4-52.9); Level 3: Beginning to take action (PAM score of 56.4-66.0); and Level 4: Has no difficulty maintaining behaviors over time (PAM score of 68.5 or above). The PAM questionnaire was translated into Hebrew and was validated [28].

### Statistical Analysis

Statistical analyses were performed using the SPSS statistical software, version 21. Chi-square tests for independence were used to examine the dependency between the duration of the site visits and sociodemographic attributes of the experienced users. Spearman correlations were used to examine the correlation between site visits and personal empowerment. Mann–Whitney and Wilcoxon nonparametric tests were used to examine the differences in PAM interval score. A Mann–Whitney test was used to examine the differences in the PAM interval scores between new and experienced users.

**Figure 1:**
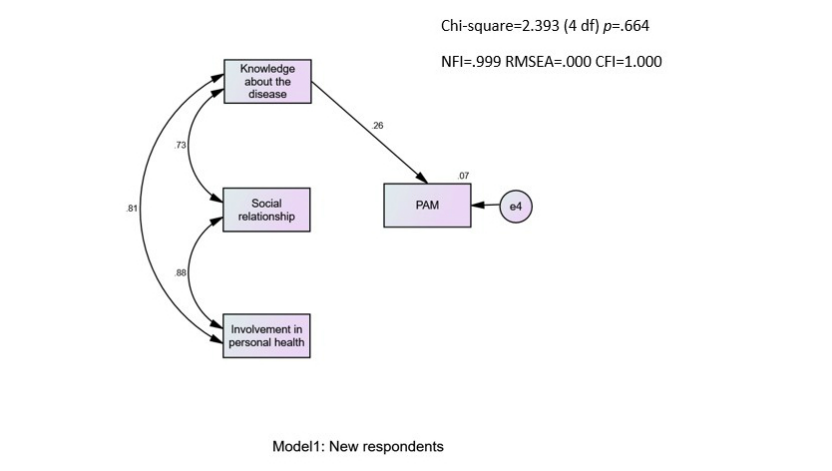
Model 1: New respondents.

**Figure 2:**
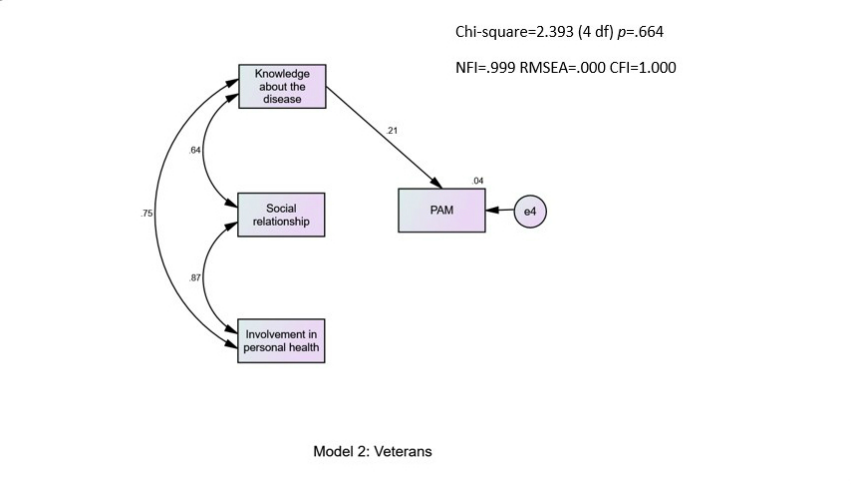
Model 2: Veteran users.

Predictors of PAM were analyzed through structural equation modeling with AMOS, version 21, creating a path analysis with the maximum likelihood method. Structural equation modeling was carried out to examine predictors of the PAM interval score. A multiple-group analysis yielded a good model fit (χ²_4_=2.4; *P*=.664; comparative fit index =1.000; normed fit index =.999; root mean square error of approximation =.000). As can be seen in Figure 1, for the new respondents, PAM was predicted directly by knowledge about the disease (β=.26) and only by that factor. Social relationship and involvement in personal health had a strong correlation with knowledge about the disease and among themselves. In Figure 2, for the experienced users, PAM level was also predicted directly by knowledge about the disease (β=.21). The correlations between the 3 factors of empowerment were also high and significant among the experienced users.

## Results

### Significant Findings

Table 1 describes the demographics of the new respondents and the experienced users.

There were several differences between the groups. New registrants were younger, included more singles, were less educated, and had lower incomes than the experienced users.

Table 2 describes the relationship between frequency and duration of visits to the Camoni site and knowledge about the disease, social relationships, and involvement in personal health among experienced users.

**Table 1:** Descriptive statistics of study variables.

Variable		New users N (%)	Experienced users N (%)	Total N (%)
Gender^a^				
	Male	123 (35.2)	154 (45.7)	277 (40.4)
	Female	226 (64.8)	183 (54.3)	409 (59.6)
Family status^a^				
	Never married	63 (18)	46 (14)	109 (16.1)
	Married	178 (52.0)	203 (60.4)	381 (56.2)
	Divorced/separated	86 (25)	62 (19)	148 (21.8)
	Widower	15 (4)	25 (7)	40 (6)
Age^a^, years				
	15-19	4 (1)	2 (0.6)	6 (0.9)
	20-39	74 (23)	28 (9)	102 (15.8)
	40-59	162 (49.2)	126 (39.9)	288 (44.7)
	60+	89 (27.1)	160 (50.6)	249 (38.6)
Education^a^				
	Elementary or high school	202 (58.6)	149 (44.2)	351 (51.5)
	Academic	143 (41.4)	188 (55.8)	331 (48.5)
Income^a^				
	Lower than average	189 (55.8)	138 (42.5)	327 (49.2)
	Average	72 (21)	85 (26)	157 (23.6)
	Above average	78 (23)	102 (31.4)	180 (27.1)
Chronic illness				
	Diabetes mellitus	96 (27)	164 (48.1)	260 (37.6)
	Pain	167 (47.7)	112 (32.8)	279 (40.4)
	Depression	129 (36.9)	68 (20)	197 (28.5)
	Hypertension	137 (39.1)	150 (44.0)	287 (41.5)
Number of illnesses				
	1	152 (53.8)	186 (58.3)	338 (49.2)
	2	106 (32.2)	96 (30)	202 (34.3)
	3+	51 (14)	37 (12)	88 (17)

^a^ The chi-square test for independence significant at *P*<.05

**Table 2:** Spearman correlations between the frequency and duration of visits and the 3 factors of personal empowerment in health, among experienced users.

Correlations	Knowledge about the disease (N=362)	Social relationships (N=374)	Involvement in personal health (N=374)
Frequency of visiting the site (1=less than once a month, 7=a few times a day)	0.251^a^	0.271^a^	0.292^a^
Duration of the visits (1=less than 10 minutes, 4=more than an hour)	0.136^a^	0.152^a^	0.190^a^

^a^*P*<.01

We found significant positive correlations between both the frequency and duration of visits to the site and personal empowerment in health. Experienced users (mean, M=69.3, standard deviation, SD=19.1, PAM level 4) had significantly higher PAM scores (*P*<.001) than did new enrollees (M=62.8, SD=18.7, PAM level 3).

Men tended to surf longer than women, as 15.8% of the men surfed more than an hour compared with 8% of women; 52% of the women surfed 10 to 30 minutes, (*P*<.05) compared with 38% of the men. Among the experienced users, 75.7% with a high school education surfed 10 minutes or more compared with only 61.2% of those with an academic education (*P*<.05).

No significant dependency was found between the experienced users’ socioeconomic level and duration of visits (*P*>.05). Experienced users with diabetes mellitus tended to surf more than experienced users with other illnesses. We found that 16.9% of the respondents with diabetes surfed for more than an hour. Only 7.1% of the respondents with other illnesses surfed for more than an hour (*P*<.05).

### Follow-Up Questionnaire

A third group was comprised of 55 respondents who answered a follow-up questionnaire approximately 3 months after the first one. They included 31 new users and 24 experienced users. Their demographics were similar to those of the new and experienced users combined. The Wilcoxon test was used to examine the interval change in the PAM score. The PAM scores increased significantly between the initial score (M=61.89, SD=20.92, PAM level 3) and the score 3 months later (M=71.08, SD=19.70, PAM level 4; Z=−3.625, *P*<.001).

Experienced respondents were asked 2 questions: what is the frequency of use of the site and the average amount of time spent surfing. A (*P*<.01) positive correlation was found between frequency and time spent and the 3 indices of health empowerment (confidence from knowledge acquired about the disease, a sense of shared support, and personal involvement in treatment).

We found significantly higher PAM scores among experienced users compared with the newly enrolled (mean 62.8 and 69.3, respectively Z=−4.197, *P*<.001). More of the experienced users (56.6%) had the highest PAM rating (level 4) across all 4 diseases (diabetes mellitus, pain, depression, and high blood pressure) compared with the newly enrolled (42.3%). Continued involvement in the site seems to be associated with increased PAM scores.

## Discussion

### Surfer Demographics

This study is unique in that we examined the association of participating in a social health network (Camoni) with patient activation, among patients with depression, diabetes mellitus, pain, or high blood pressure. The main findings among Camoni surfers indicate that those with at least 6-month experience on the site had the highest PAM (level 4) compared with new visitors (level 3; Z=−3.938, *P*<.00). Among those who participated an additional 3 months, 54.5% reached level 4 PAM scores.

We found that more patients with chronic diseases surfing on Camoni were from lower socioeconomic groups (less educated, lower income) and were both passively and actively using the social health network site compared with those with higher education and income levels. Other studies have reported inconsistent results regarding factors such as income and age with Web-based health-related information seeking [29-31]. As computer access increases over time, lower income groups are increasingly using the Internet [32]. The greatest growth is seen among those who have low incomes and are less educated. This is particularly important because lower socioeconomic groups often have lower patient activation [4,14,33-34] and poorer health outcomes. In the present study, we also saw that the social health network seemed to benefit lower income and less-educated participants.

In our sample, more men used the site than did women. Although earlier studies reported that women were more likely to use health and general social network sites [4], more recent data indicate that this trend is changing. A 2014 Pew survey found that men and women use social networking sites equally [35].

We also found that individuals ages 40 to 59 years were more likely to use the social network site (both new and experienced users) than their younger counterparts did. Prior studies reported that older people use the Internet less and that older generations prefer to wait for a physician consultation [17,34,36]. However, older adults are increasingly likely to turn to the Internet and social networking sites [37]. Increasing computer literacy has the potential to increase patient well-being greatly. We know that 75% of adults ages 65 years and older are living with a chronic condition [33]. As the population continues to age, more and more elderly will likely turn to social networks for support and information, thus improving patient activation and well-being.

### Duration of Visit

We found that increased frequency and duration of visits to the social network were associated with increased PAM scores. Those who surf more frequently and for longer times had higher scores on 3 empowerment measurements: patient feels confidence in acquired knowledge about the disease; feelings of support and the ability to share their experience; and level of involvement in treatment.

### Disease and Health Knowledge

Specific health groups turn to the social health network for several reasons [4-10], and most respondents want to obtain information and are not seeking social relationships [12-15]. They expect the website to be specifically directed to their chronic health condition, and they seek knowledge about the disease and about treatment options. Likewise, we found that knowledge had the greatest influence on patient activation. This is consistent with the literature on patient activation and the assumption that consumers will make more prudent health and health care choices and will increase their “activation” when they are given relevant and quality information [9]. Several recent studies considered the role of Internet use and patient activation in the chronically ill [38-40] as well as in the general population [41]. Those who access health information over the Internet are more likely to have higher PAM.

No difference was found in PAM levels between those who post on the site and lurkers (those who merely observe but do not post). We suggest that this is further evidence of the importance of knowledge acquisition over the social element. This finding is similar to that of other studies that reported that those who posted more frequently were no more likely to be activated or empowered than lurkers were [42]. It is most likely that simply reading and searching for information within the social network site was sufficient to increase activation levels [43] and that obtaining information is a key factor.

We suggest that the finding that respondents with diabetes mellitus particularly benefited from the website is related to the increased importance of self-care and knowledge for patients with diabetes. Most of the day-to-day care for diabetic patients is carried out by the patients and their families. People with diabetes in particular require active participation in their medical care [44]. Patients need to monitor blood glucose levels, adjust insulin doses exercise, and monitor their diet with consequences that are often immediate. They therefore may be more inclined to seek information and peer support and become more involved in their health care.

### Limitations of this Study

This is a cross-sectional study, and an association is not necessarily causation. Longitudinal studies are needed to determine the arrow of causation. It may be that of continued participation in a social health network leads to increased patient activation, or that increased patient activation facilitates participation in a social health network.

Another limitation of the study arises from the sampling method. The study population was self-selected, and therefore, the results may be biased. It included only Israelis, was conducted in the Hebrew language, and it probably did not include new immigrants or the non-Jewish population. It is possible that people with certain characteristics tend to respond to Web-based surveys. We cannot know who chose not to participate in the Web-based questionnaire and whether the results would have been the same had they participated. Also, it is possible that those who actively participated and those who were passive participants (lurkers) differed. Another limitation was that we did not analyze the different diseases separately, according to the type of knowledge and support provided, although all participants had similar exposure to the contents of the site. We did not have information about other sources of knowledge or support that the participants might have accessed, such as additional medical consultations, family members who provided support, other forums, or Internet sites where they received information, or avenues such as medical books or pamphlets. Also, only 4 of the 16 communities were selected. Perhaps other diseases would have led to different results. In addition, we resurveyed the users 3 months after registration. We cannot know how many times they actually entered the site compared with what they reported. Had we conducted the second survey after a longer period, the results might have been different.

Finally, response to the follow-up questionnaire was lower than expected. This finding is similar to that of others who described [45] similar poor follow-up on Internet questionnaires.

Such limitations are expected in all studies among anonymous, surfing patients. As a result, these types of studies describe trends and attitudes and probably represent an iceberg phenomenon of social networks.

### Conclusions

Among patients with a chronic illness, surfing on an Internet-based, health-related social network was associated with improved knowledge and health activation. This was stronger among those with less education, lower income, men, older age, and patients with diabetes.

Knowledge about one’s disease had a direct effect on patient activation in a structural equation model. Social health networks seem to provide an opportunity for people from different strata to increase activation by increasing knowledge about their disease and about their personal health.

## Abbreviations

M: mean
PAM: Patient Activation Measure
SD: standard deviation
